# Perioperative, Functional, and Oncological Outcomes of Robotic-Assisted Partial Nephrectomy in 1,267 Indian Patients: A Multicenter Analysis by the Indian Robotic Partial Nephrectomy (IRPN) Collaborative Group

**DOI:** 10.7759/cureus.84209

**Published:** 2025-05-16

**Authors:** Gagan Gautam, Arvind Ganpule, Anant Kumar, Mohan Keshavamurthy, Sudhir Rawal, Ravimohan Mavuduru, Kishore TA, Ginil kumar Pooleri, Deepak Dubey, Narasimhan Ragavan, Hemang Bakshi, Sanjai Addla, SK Raghunath, Divya Gupta, Kamal Malik, Akhil Dahiya

**Affiliations:** 1 Department of Uro-Oncology and Robotic Surgery, Medanta - The Medicity, Gurugram, IND; 2 Urology, Muljibhai Patel Urological Hospital, Nadiad, IND; 3 Uro-Oncology, Robotic, and Kidney Transplantation, Max Super Speciality Hospital, Saket, New Delhi, IND; 4 Urology, Uro-Oncology, Andrology, Transplant, and Robotic Surgery, Fortis Hospital, Bannerghatta Road, Bengaluru, IND; 5 Department of Genito Uro-Oncology Services, Rajiv Gandhi Cancer Institute and Research Centre, Delhi, IND; 6 Urology, Postgraduate Institute of Medical Education and Research, Chandigarh, IND; 7 Department of Urology, Aster Medcity, Kochi, IND; 8 Urology, Amrita Institute of Medical Sciences, Kochi, IND; 9 Department of Urology, Manipal Hospitals, Bengaluru, IND; 10 Urology, Apollo Hospitals, Chennai, IND; 11 Department of Urology, HCG Cancer Center, Ahmedabad, IND; 12 Robotic Uro-Oncology, Apollo Hospitals, Hyderabad, IND; 13 Department of Uro-Oncologyand Robotic Surgery, HCG Hospitals, Bengaluru, IND; 14 Clinical Operations, Catalyst Clinical Services Pvt. Ltd., Delhi, IND; 15 Management, Intuitive Surgical, Sunnyvale, USA; 16 Clinical and Medical Affairs, Intuitive Surgical, Sunnyvale, USA

**Keywords:** minimal access surgery, partial nephrectomy, robotic-assisted surgery, t1a renal masses, t2 renal masses

## Abstract

Introduction: Robotic-assisted partial nephrectomy (RPN) is increasingly recognized as an effective treatment for small renal masses. This study aims to highlight the therapeutic benefits of RPN for both small and relatively larger renal masses in the Indian population.

Methods: A retrospective chart review was conducted on patients who underwent RPN using the da Vinci surgical system between September 2010 and September 2022 across 14 centers located in various cities of India, including Ahmedabad, Bengaluru, Chandigarh, Chennai, Delhi, Gurugram, Hyderabad, Kochi, and Nadiad. Data on demographics, medical history, clinical characteristics, and perioperative, functional, and oncological outcomes were extracted from medical records and analyzed statistically.

Results: A total of 1,267 patients were included in the study, with 757 in the T1a tumor group and 510 in the T1b+T2 tumor group. In terms of baseline characteristics, the two groups showed a significant difference (p < 0.001) in renal nephrometry score (RENAL score). The mean operating room time (201.31 ± 77.57 vs. 191.06 ± 74.51; p = 0.0021) and warm ischemia time (25.21 ± 8.08 vs. 22.51 ± 7.95; p < 0.001) were significantly higher in the T1b+T2 tumor groups. Other outcomes were comparable, namely, length of hospital stay (4.21 ± 2.47 vs. 4.05 ± 2.30 days; p = 0.2459), postoperative complications (3.33% vs. 2.11%; p = 0.181), conversion rates (0% vs. 0%), and surgical margins (3.04 vs. 4.31%, p = 0.229). There was no difference in recurrence rates, and no significant differences were observed in the functional outcomes between the two groups.

Conclusion: RPN provides encouraging surgical, oncological, and functional outcomes for both T1a and T1b+T2 renal masses, enabling nephron-sparing surgery and early recovery of renal function.

## Introduction

The management of localized renal tumors has evolved considerably over the past two decades. Partial nephrectomy (PN) is now the gold standard for small renal masses, especially T1a tumors (<4 cm), owing to its ability to preserve renal function without compromising oncological outcomes [1]. Major urological associations recommend PN as the preferred approach for T1a and, when feasible, T1b tumors [2-4]. PN is associated with reduced long-term risk of chronic kidney disease (CKD), cardiovascular morbidity, and improved overall survival compared to radical nephrectomy (RN) [5,6]. While PN was historically reserved for smaller tumors, increasing expertise and improved techniques have expanded its application to more complex tumors, including selected T2 lesions in patients with solitary kidneys, bilateral tumors, or underlying renal dysfunction [7,8]. Open partial nephrectomy (OPN), despite its effectiveness, is invasive and involves longer recovery times. By contrast, laparoscopic partial nephrectomy (LPN) offers a minimally invasive alternative with reduced morbidity. However, LPN demands advanced technical skill, particularly for intracorporeal suturing and tumor dissection, which has limited its widespread adoption [9,10].

The introduction of robot-assisted partial nephrectomy (RPN) by Gettman et al. in 2004 [11] transformed nephron-sparing surgery. RPN combines the benefits of laparoscopy with enhanced dexterity, 3D magnification, and tremor filtration. These features improve tumor excision and facilitate rapid renorrhaphy, translating to shorter warm ischemia time (WIT) and better preservation of renal function [12-14]. The adoption of RPN has grown rapidly, with its usage increasing from 21% in 2009 to 58% in 2015 in the United States [15]. A growing body of evidence has confirmed RPN’s superiority over LPN in terms of lower blood loss, fewer conversions to open surgery, shorter hospital stays, and reduced postoperative complications [16-18]. Furthermore, RPN is associated with a shorter learning curve, making it more accessible to urologists transitioning from open or laparoscopic approaches [19]. Emerging data have also shown the feasibility of RPN in managing larger (>7 cm), complex (endophytic, hilar, cystic), or T2 tumors, with acceptable oncological and functional outcomes [20-22]. However, many of these studies are limited to single-center or high-volume institutions in Western countries. As such, the findings may not be generalizable to low- and middle-income countries, where anatomical and demographic variables differ significantly. Evaluating RPN in T1b and T2 tumors is essential to support the expanding role of nephron-sparing surgery in larger tumors, especially in patients with imperative indications like solitary kidneys, bilateral tumors, or chronic kidney disease. It also helps determine if RPN can deliver oncologic outcomes comparable to radical surgery while preserving renal function. Long-term oncological outcomes following RPN, especially in T1b and T2 tumors, remain underexplored. 

A systematic review by Campbell et al. (2021) noted a lack of robust, prospective data comparing long-term cancer-specific survival and recurrence across surgical techniques for higher-stage renal tumors [2]. Moreover, several reviews have highlighted the absence of stratification by tumor type in comparisons between open, laparoscopic, and robotic PN [23]. Importantly, ethnic and regional differences can influence surgical outcomes. For instance, Indian patients often present with higher perinephric fat thickness and delayed diagnoses, which contribute to increased tumor complexity [24,25]. These anatomical features may hinder dissection and increase operative time and complications during RPN. However, despite these challenges, data on RPN from the Indian subcontinent remains sparse, with most reports being small, single-institution case series. 

This study addresses a critical evidence gap by evaluating RPN outcomes specifically within the Indian population, a demographic underrepresented in existing literature. Ethnic and anatomical factors, such as increased perinephric fat thickness, which is common in Indian patients, may influence surgical complexity and outcomes. To our knowledge, this is the largest multicenter study in India to date focused on RPN in both T1 and selected T2 renal tumors. By offering real-world insights into the safety, efficacy, and perioperative advantages of RPN in diverse clinical scenarios, this study not only strengthens the case for its broader adoption but also sets the foundation for region-specific surgical guidelines. The findings are poised to inform evidence-based clinical decision-making and advance the surgical management of renal tumors in the Indian context.

## Materials and methods

This retrospective, multicenter real-world study was conducted across 14 Indian centers. Patient charts were reviewed for those who underwent RPN using the Da Vinci Surgical System (Intuitive Surgical, Sunnyvale, CA, USA) between September 2010 and September 2022. The inclusion criteria were male and female patients aged 18 years and older who had undergone open, laparoscopic, or robotic-assisted PN. The exclusion criteria included subjects with more than two masses in the affected kidney requiring multiple partial nephrectomies, a solitary or horseshoe kidney, a history of any prior surgery on the affected kidney, excluding endoscopic kidney stone surgery within the past year, and those scheduled for simultaneous bilateral PN. No formal sampling technique was used; instead, all patients who met the eligibility criteria during the study period were included. The study adhered to the latest Helsinki Declaration and Good Clinical Practice guidelines. The study was conducted across 14 centers located in various cities of India, including Ahmedabad, Bengaluru, Chandigarh, Chennai, Delhi, Gurugram, Hyderabad, Kochi, and Nadiad. Prior to initiating the study, ethics committee approval was obtained at each center, and the study was registered with the Clinical Trials Registry of India vide registration number CTRI/2022/04/041924.

Demographic data, medical history, clinical characteristics, and perioperative, functional, and oncological outcomes were extracted from patient records. Perioperative and postoperative outcomes, including operating room time, estimated blood loss (EBL), WIT, hospital stay, creatinine, eGFR, complications, malignancy status, resection margins, and tumor size, were also recorded. The Clavien-Dindo classification was used to grade complications. From the time of discharge up to one year later, functional outcomes were collected. Oncological outcomes were assessed at one, two, three, four, and five years post-surgery. T1a tumors were compared to T1b+T2 tumors in terms of perioperative, functional, and oncological outcomes. The continuous variables were summarized as arithmetic means with standard deviation (SD), while categorical and nominal data were presented as frequencies and percentages. The independent sample t-test analyzed continuous data between two groups, and the Wilcoxon rank-sum test compared medians between the two groups. Pearson's chi-square test or Fisher’s exact test assessed frequency differences. A two-sided p < 0.05 was considered statistically significant. Statistical analysis was conducted using Stata IC 13.1 (StataCorp LLC, Texas, USA).

## Results

Baseline characteristics of the participants

The study included data from 1,267 patients, with 757 in the T1a tumor group and 510 in the T1b+T2 tumor group who underwent RPN. Preoperative descriptive characteristics are summarized in Table 1.

**Table 1:** Descriptive characteristics of the preoperative variables of Indian robotic partial nephrectomy patients *Significant value. ^¤^ Data available for 561 patients in the T1a group and 367 patients in the T1b+T2 group. ^¥^ Data available for 605 patients in the T1a group and 427 patients in the T1b+T2 group. SD: standard deviation; CAD: coronary artery disease; COPD: chronic obstructive pulmonary disease; eGFR: estimated glomerular filtration rate

Variable	T1a (N = 757)	T1b+T2 (N = 510)	p-value	t-value/χ² value
Age, mean ± SD, year	53.16 ± 12.81	53.43 ± 13.16	0.7125	-0.3686
Sex, n (%)				
Male	531 (70.15)	366 (71.76)	0.534	0.3865
Female	226 (29.85)	144 (28.24)		
Comorbidities, n (%)				
Hypertension	337 (44.52)	235 (46.08)	0.584	0.2997
Congestive heart failure	7 (0.92)	3 (0.59)	0.507	0.4405
CAD	30 (3.96)	23 (4.51)	0.634	0.2273
Peripheral vascular disease	1 (0.13)	0	0.412	0.6742
COPD	18 (2.38)	11 (2.16)	0.796	0.0665
Diabetes	219 (28.93)	131 (25.69)	0.205	1.6036
Chronic kidney disease	130 (17.17)	93 (18.24)	0.626	0.2371
Liver disease	14 (1.85)	6 (1.18)	0.346	0.8882
Chronic steroid/immunosuppressant use	2 (0.26)	1 (0.20)	0.807	0.0599
Metastatic solid tumor	4 (0.53)	3 (0.59)	0.888	0.0199
Previous abdominal surgery (in the past one year), n (%)	13 (1.72)	5 (0.98)	0.277	1.1815
Side, n (%)				
Left	358 (47.29)	273 (53.53)	0.029*	4.7421
Right	399 (52.71)	237 (46.47)	0.029*	4.7421
Tumor location, n (%) ^¤^				
Anterior	232 (41.35)	137 (37.33)	0.146	2.1143
Posterior	200 (35.65)	142 (38.69)	0.576	0.3131
Hilar	33 (5.88)	21 (5.72)	0.835	0.0436
Other	96 (17.11)	67 (18.26)	0.812	0.0564
Creatinine, mean ± SD, mg/dl	0.92 ± 0.35	0.98 ± 0.52	0.0533	-1.9343
eGFR, mean ± SD, ml/min	91.90 ± 26.33	90.86 ± 30.54	0.5742	0.5620
Renal nephrometry score categories, n (%)^ ¥^				
Low (≤6)	324 (53.55)	119 (27.87)	<0.001*	50.7830
Intermediate (7-9)	224 (37.02)	216 (50.59)	<0.001*	21.8954
High (≥10)	57 (9.42)	92 (21.55)	<0.001*	32.4323

Significant differences were noted between the groups in terms of tumor laterality (p = 0.029), with right-sided tumors being more common in the T1a group and left-sided tumors predominating in the T1b+T2 group. In addition, RENAL scores differed significantly (p < 0.001), with the T1a group having more patients in the low-score category, while the T1b+T2 group had a higher proportion of patients with intermediate and high scores. All other demographic and preoperative variables were comparable between the two groups. While the multicenter design enhances generalizability by including diverse populations and practices, variability in surgeon experience and learning curves, factors not accounted for in this study, may have influenced the outcomes.

Intraoperative variables of the study population

Table 2 summarizes the intraoperative and postoperative variables.

**Table 2:** Intra- and postoperative variables of Indian robotic partial nephrectomy patients *Significant value SD: standard deviation; IQR: interquartile range

Variables	T1a (N = 757)	T1b+T2 (N = 510)	p-value	t-value/χ² value
Operating room time, mean ± SD, min	191.06 ± 74.51	201.31 ± 77.57	0.0221*	-2.2911
Length of hospital stay, mean ± SD, days	4.05 ± 2.30	4.21 ± 2.47	0.2459	-1.1609
Warm ischemia time, min				
Mean ± SD	22.51 ± 7.95	25.21 ± 8.08	<0.001*	-5.8797
Intraoperative complications, n (%)	16 (2.11)	8 (1.57)	0.485	0.4870
Clavien-Dindo classification				
Grade I	13 (81.25)	5 (62.50)	0.302	-
Grade II	2 (12.50)	2 (25.00)	0.407	-
Grade III	1 (6.25)	1 (12.50)	0.565	-
eGFR, mean ± SD, ml/min (immediate postoperative period)	82.17 ± 26.44	79.62 ± 28.13	0.1348	1.4966
Conversion to open partial nephrectomy, n (%)	0	0	-	-
Malignant tumor on biopsy, n (%)	689 (91.02)	467 (91.57)	0.733	0.1159
Tumor size, mean ± SD, mm	29.78 ± 7.52	55.03 ± 21.73	-	-
Positive surgical margin, n (%)	23 (3.04)	22 (4.31)	0.229	1.4470
Postoperative complications (post discharge till 90 days post-surgery), n (%)	16 (2.11)	17 (3.33)	0.181	1.7871
Clavien-Dindo classification				
Grade I	13 (81.25)	15 (88.24)	0.470	-
Grade II	1 (6.25)	2 (11.76)	0.523	-
Grade III	2 (12.50)	0	0.227	-

**Figure 1:**
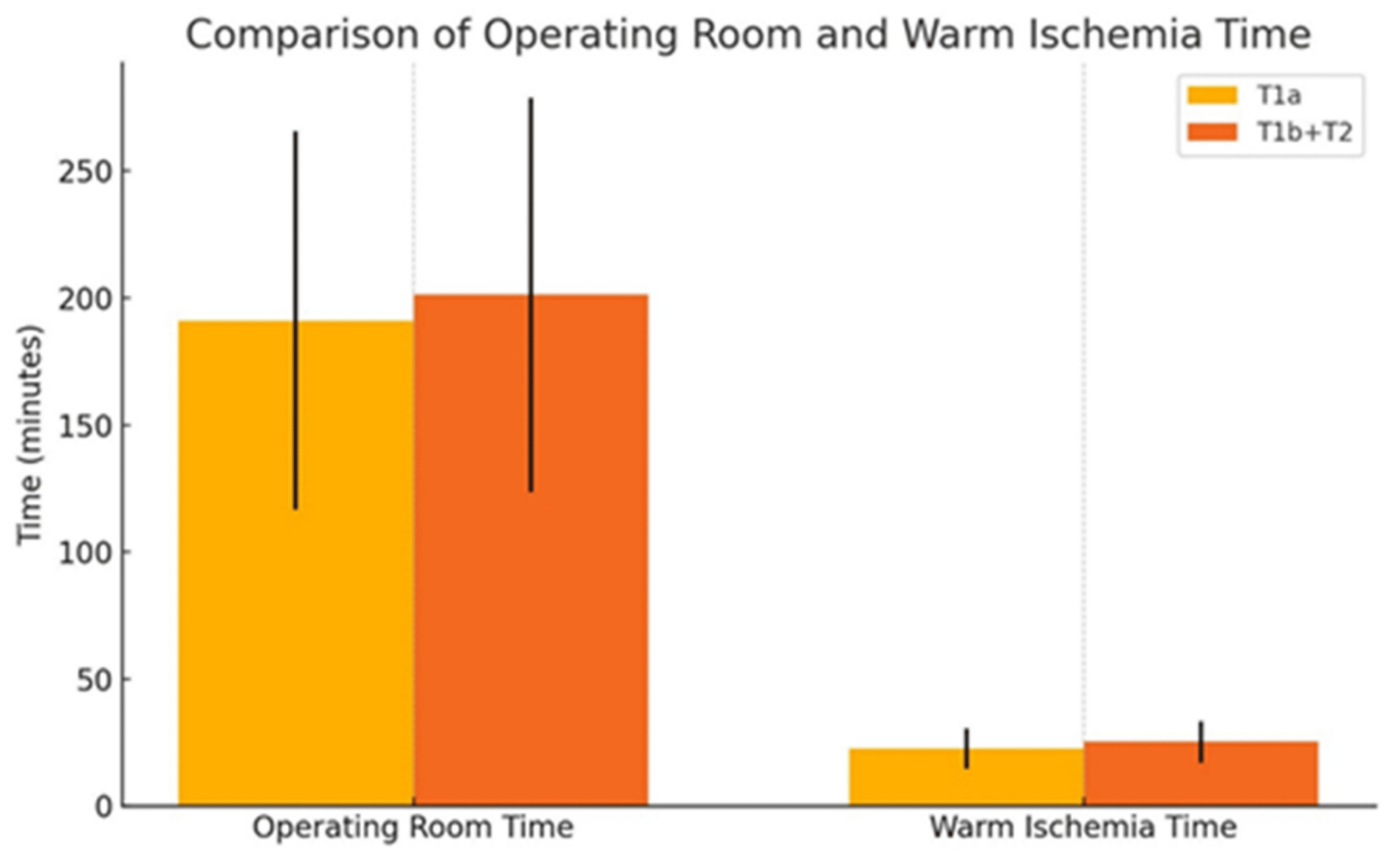
Operating room time and warm ischemia time for the T1a and T1b+T2 groups

The T1b+T2 tumor group had significantly higher mean operating room time (201.31 ± 77.57 vs. 191.06 ± 74.51; p = 0.0221) and WIT (25.21 ± 8.08 vs. 22.51 ± 7.95; p < 0.001). Figure 1 illustrates the comparison of operating room time and warm ischemia time between the T1a and T1b+T2 groups. 

Hospital stay was comparable between the T1b+T2 and T1a groups (4.21 ± 2.47 vs. 4.05 ± 2.30 days; p = 0.2459). The intraoperative complication rate was comparable (1.57% vs. 2.11%; p = 0.485). No cases required conversion to open PN in either group. Malignancy was found in over 90% of patients in both groups. There were no significant differences in tumor resection margins (4.31% vs. 3.04%; p = 0.229).

Short-term and long-term follow-up data of the study population

The short-term and long-term functional and oncological outcomes are summarized in Tables 2-4.

**Table 3:** Change in renal functions following robotic partial nephrectomy in Indian patients SD: standard deviation; eGFR: estimated glomerular filtration rate

Variables	Preoperative		Postoperative days 1-7		Postoperative one year	
	T1a	T1b+T2	T1a	T1b+T2	T1a	T1b+T2
Creatinine, mean ± SD, mg/dl	0.92 ± 0.35	0.98 ± 0.52	1.04 ± 0.38	1.12 ± 0.56	0.96 ± 0.31	1.04 ± 0.53
% change in creatinine compared to preoperative values (mean % change)			13.04%	14.29%	4.35%	6.12%
eGFR, mean ± SD, ml/min	91.90 ± 26.31	90.86 ± 30.50	82.17 ± 26.42	79.62 ± 28.10	89.33 ± 26.05	85.55 ± 27.63
% change in eGFR compared to preoperative values (mean % change)			-10.59%	-12.37%	-2.80%	-5.84%

**Table 4:** Oncological outcomes of the study population following robotic partial nephrectomy in Indian patients

Follow-up variables	T1a	T1b+T2	p-value	t-value/χ² value
One year	N = 400	N = 302		
Recurrence, n (%)	6 (1.50)	6 (1.99)	0.417	-
Location of recurrence, n (%)				
Lung	1 (0.25)	1 (0.33)		
Lymph nodes	1 (0.25)	1 (0.33)		
Same kidney	3 (0.75)	1 (0.33)		
Any other location	1 (0.25)	1 (0.33)		
Opposite kidney	0	1 (0.33)		
Same kidney, bones	0	1 (0.33)		
Two years	N = 205	N = 148		
Recurrence, n (%)	1 (0.49)	1 (0.68)	0.663	-
Location of recurrence, n (%)				
Same kidney	1 (0.49)	0		
Opposite kidney	0	1 (0.68)		
Three years	N = 89	N = 77		
Recurrence, n (%)	0	2 (2.60)	0.214	-
Location of recurrence, n (%)				
Lung	0	1 (1.30)		
Same kidney	0	1 (1.30)		
Four years	N = 39	N = 26		
Recurrence, n (%)	1 (2.56)	1 (3.85)	0.644	-
Location of recurrence, n (%)				
Same kidney	1 (2.56)	0		
Lung	0	1 (3.85)		
Five years	N = 15	N = 11		
Recurrence, n (%)	0	1 (9.09)	0.423	-
Location of recurrence, n (%)				
Same kidney	0	1 (9.09)		

**Figure 2:**
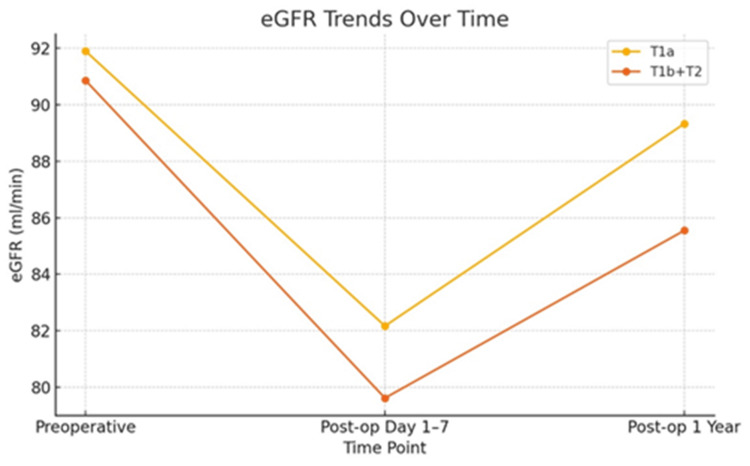
eGFR trends over time for the T1a and T1b+T2 groups

Most patients (>95%) had no complications during short-term follow-up (discharge to 90 days), and the postoperative complication rates were comparable (3.33% vs. 2.11%; p = 0.181). The mean creatinine and eGFR remained within the normal range at postoperative days 1-7 and at the one-year follow-up. Figure 2 illustrates the trend of eGFR values across different time points in the T1a and T1b+T2 groups. 

The percentage change in creatinine and eGFR at postoperative days 1-7 and one year was non significantly higher in the T1b+T2 tumor group. However, functional and oncological outcomes were comparable between groups, with a total of 19 recurrences (8 in the T1a group and 11 in the T1b+T2 group). Due to the smaller number of events, the median disease-free survival (DFS) was not estimable for either group. Recurrence rates at one, two, three, four, and five years were 1.50%, 0.49%, 0%, 2.56%, and 0% for the T1a group and 1.99%, 0.68%, 2.60%, 3.85%, and 9.09% for the T1b+T2 group, respectively.

## Discussion

RPN, initially the standard for small renal masses, has expanded to include large and complex tumors. Advances in robotic techniques allow kidney preservation while maintaining the benefits of minimally invasive surgery. Although observational studies highlight RPN’s effectiveness for complex renal masses, most are single-center reports with limited data. To address this gap, we conducted a large multi-institutional study comparing the functional, oncologic, perioperative, and postoperative outcomes of RPN in T1a and T1b+T2 renal masses. The role of PN in T1a tumors is well established, and global guidelines advocate its use for T1b tumors due to its nephron-sparing surgery benefits and oncologic efficacy [26]. Population-based data suggest PN and RN offer comparable cancer control for T1b tumors, although PN use varies by region [4,27]. PN has also shown success in high-risk tumors (>7 cm) with acceptable outcomes [28]. While RN remains standard for T2 tumors, emerging evidence supports PN in select cases [27,29]. Some studies suggest PN does not impact cancer-specific mortality in T2 tumors, while others report higher mortality for PN in tumors >7 cm [30,31]. As a result, the role of PN in larger tumors remains under investigation. The optimal approach for PN prioritizes complete tumor removal, minimal complications, and renal function preservation. Robotic surgery offers advantages such as smaller incisions, limited anatomical exposure with reduced risk of adjacent organ damage, and postoperative complications. A meta-analysis found that RPN had lower morbidity and better renal function preservation than open surgery [32]. Another review suggested that PN is preferable to RN for T1b tumors (>4 cm) and that RPN overcomes laparoscopic PN’s limitations [33]. With enhanced visualization and precise instrumentation, RPN addresses laparoscopic challenges in tumor dissection. A meta-analysis confirmed RPN as a viable alternative with shorter WIT [34].

In our study, the mean operating time was 191.06 ± 74.51 minutes for T1a tumors and 201.31 ± 77.57 minutes for T1b+T2 tumors (p = 0.0221). The procedure length is influenced by factors such as surgeon experience, tumor size, and location. A study on RPN for 2.4 cm tumors reported an operative time of 190 minutes [35]. A study comparing perioperative outcomes in RPN patients found that operating times differed for imperative versus elective cases (186 vs. 180 minutes; p = 0.55) [36]. Another study reported a mean operative time of 182.5 ± 68.6 minutes [37], while a study with a mean preoperative tumor size of 33 mm found an operative time of 156.3 minutes [38]. Surgical duration can be extended due to the need for patient repositioning or robotic docking adjustments. In our study, the mean operating time for T1a tumors aligned with previous reports, while T1b+T2 tumors required longer due to their complexity.

WIT is a crucial factor in PN success, as each additional minute is linked to a 6% higher risk of acute renal failure, a 7% increased risk of acute-onset end-stage renal disease (ESRD), and a 4% higher risk of new-onset ESRD, independent of preoperative renal function, tumor size, and surgical approach [39]. Achieving the "Trifecta" requires maintaining WIT below 25 minutes [40]. Tumor complexity influences WIT, which in turn affects renal function, with longer WIT associated with poorer renal preservation [41]. In our study, the mean WIT was 22.51 ± 7.95 minutes for T1a tumors and 25.21 ± 8.08 minutes for T1b+T2 tumors (p < 0.001). In corroboration, a previous study reported a WIT of 21 minutes (range 0-55) in patients undergoing RPN with a PADUA score of ≥10 [41]. A recent evidence-based study found that RPN achieves an acceptable WIT for complex renal tumors (RENAL score ≥7), with reported mean WITs ranging from <25 minutes to 28 minutes [42]. Some studies observed significantly higher WIT for tumors >4 cm (T1b) compared to ≤4 cm (T1a), such as 24 versus 17 minutes (p < 0.001) [43] and 25 versus 20 minutes (p = 0.011) [38,41,44]. Our findings align with these previously published reports.

The mean RENAL score for T1a and T1b+T2 tumors in our study was ≤6 (low) in 53.55% and 27.87% of patients, 7-9 (intermediate) in 37.02% and 50.59% of patients, and ≥10 (high) in 9.42% and 21.55% of patients. Kopp et al. suggested that a RENAL score of ≥10 is negatively associated with OS in T2 masses compared to a score of <10 and that PN may provide oncological benefits for T2 renal masses [45]. Furthermore, for T2 masses with a RENAL sum ≤10, but not >10, RN is independently linked to a decline in renal function compared to PN, with a greater relative decrease in eGFR for each unit decrease in the RENAL sum [46]. A recent systematic review and meta-analysis of 22 studies found no significant differences between RPN and laparoscopic PN in any perioperative outcomes for moderate- to high-complexity renal masses (RENAL and PADUA score ≥10). In addition, in these complex cases, RPN was associated with significantly lower EBL, complication rates, and transfusion requirements compared to open PN [47]. Other studies suggest that RPN is effective and achieves optimal outcomes in most patients, regardless of tumor complexity [38,41,42].

In our study, the mean length of hospital stay was 4.05 ± 2.30 days for T1a tumors and 4.21 ± 2.47 days for T1b+T2 tumors (p = 0.2459). Consistent with our findings, a propensity-matched analysis demonstrated a shorter hospital stay for RPN compared to open PN (4.4 vs. 6.3 days; p < 0.0001) and laparoscopic PN (4.2 vs. 6.2 days; p < 0.0001) in matched cohorts [48]. Negative surgical margins are crucial for achieving the Trifecta in PN [40]. In our study, positive margins were 3.04% for T1a tumors and 4.31% for T1b+T2 tumors (p = 0.229). A systematic review linked positive margins to increased local recurrence, recurrence-free survival, and metastasis-free survival but found no impact on cancer-specific or OS. Reported positive margin rates for RPN vary, including 7% overall [49], 3.9% for moderate to highly complex tumors, and 5.2% for high complexity cases [47]. Other studies report 4.3% [16], 2.5% in T1b tumors [50], and 0-3.7% for RENAL scores >9 [51]. Our findings align with these acceptable rates.

When choosing between RN and PN, preserving renal function remains a key consideration. A single-institution study on clinical T1b kidney cancer found RN led to a 25% increased risk of cardiac death and a 17% higher all-cause mortality rate [52]. PN for T1b tumors was associated with better OS and postoperative renal function while maintaining comparable cancer-specific and progression-free survival rates to RN [53]. A meta-analysis showed PN reduced the risk of severe CKD by 61% and all-cause mortality by 19% [54]. In our study, the decrease in eGFR for T1a tumors over seven days and one year was -10.59% and -2.80%, respectively, while for T1b+T2 tumors, it was -12.37% and -5.84%. Similarly, creatinine increased by 13.04% and 4.35% for T1a and by 14.29% and 6.12% for T1b+T2 over the same periods. Other studies have shown that RPN leads to a smaller decline in eGFR than open or laparoscopic PN, with reduced CKD upstaging and a higher five-year CKD-free survival rate [37,48]. In addition, RPN had lower acute kidney injury rates, likely due to shorter ischemia times and selective arterial clamping. For complex tumors (PADUA ≥10), post-RPN creatinine levels remained stable, aligning with other reports [55,37]. In our study, the mean creatinine and eGFR remained within normal ranges, with no significant differences in functional or oncological outcomes between T1a and T1b+T2 tumors.

Future research should focus on prospective, multicenter studies with standardized surgical and perioperative protocols to reduce variability and better evaluate the true impact of RPN on outcomes. Long-term oncological follow-up is essential to assess recurrence-free survival, cancer-specific survival, and overall survival, particularly in patients with T1b+T2 tumors. In addition, future work should examine the influence of surgeon experience, learning curves, and institutional volume on surgical outcomes, which could help inform training and credentialing practices.

Limitations

Our study has some limitations. Variability in surgeon involvement may have influenced results, although it enhances external validity compared to single-center studies. In addition, differences in surgeon learning curves were not accounted for, and the retrospective design carries inherent limitations. While a prospective randomized study on RPN for varying tumor complexities is ideal, existing data provide strong evidence supporting the role of robotic surgery in the Indian context. Furthermore, the absence of experimental validation, limited methodological detail, and dependence on a relatively narrow dataset may impact the generalizability and reproducibility of the study's findings.

## Conclusions

Single-center studies with small sample sizes often limit the reliability of conclusions, leading to inconsistent results. This large multi-institutional study strengthens the evidence supporting RPN for small and relatively larger renal masses. Despite its complexity, RPN can achieve favorable surgical, oncological, and functional outcomes for both T1a and T1b+T2 tumors when performed by experts. The role of RPN in managing complex cases, particularly larger T2 tumors, continues to be debated. However, in well-selected patients, RPN may serve as a viable alternative to RN. Clinicians should consider RPN for patients with localized T2 tumors who have favorable tumor characteristics (e.g., exophytic location, low RENAL score components) and adequate renal function and are managed by experienced surgical teams. Close postoperative surveillance, including periodic imaging and renal function assessment, is essential to ensure oncological control and nephron preservation.
